# Initial Translation of a Dementia Caregiver Intervention Into a Mobile Health Application

**DOI:** 10.1093/geroni/igaa057.877

**Published:** 2020-12-16

**Authors:** Taylor Maynard, Travis Frink, Kunal Mankodiya, Jennifer Davis, Brian Ott, Lisa Uebelacker, Geoffrey Tremont

**Affiliations:** 1 Rhode Island Hospital, Providence, Rhode Island, United States; 2 University of Rhode Island, Kingston, Rhode Island, United States; 3 University of British Columbia, Kelowna, British Columbia, Canada; 4 Butler Hospital, Providence, Rhode Island, United States

## Abstract

Caring for a person with dementia is associated with negative outcomes. Few caregiver interventions have been implemented in community settings. Mobile technology is one method for reaching many caregivers. This project translated two empirically-supported interventions for dementia caregivers into a mobile health application. A team of clinical researchers and computer engineers developed an App called CARE-Well (Caregiver Assessment, Resources, and Education) over 6 months. The group worked closely to do the following: 1). translate interventional content to be compatible with a mobile platform; 2). create new materials; 3). determine App components that captured key intervention areas; 4). troubleshoot formatting, technology, and data security; and 5). educate each other about respective areas of expertise. We developed a beta version of the App that included: 1). assessment of caregiver stress and care recipient behavioral problems; 2). psychoeducation; 3). goal diary; 4). managing behavior problems; 5). online message forum; and 6). video library. Several challenges arose during the App development process, such as how to create navigation paths and goal lists based off users’ assessment responses, data storage and usage tracking, enlarging text, and how to ensure privacy and confidentiality in the online message forum. Our experience developing the CARE-Well App showed that translating behavioral interventions into mobile health applications is feasible and dependent upon regular communication among multidisciplinary team members. Next steps for the App include beta testing with dementia caregivers and conducting a pilot randomized trial to determine feasibility for a future trial and its effects on caregiver stress.

